# High Biofilm Formation of Non-Smooth *Candida parapsilosis* Correlates with Increased Incorporation of GPI-Modified Wall Adhesins

**DOI:** 10.3390/pathogens10040493

**Published:** 2021-04-19

**Authors:** Ana Esther Moreno-Martínez, Emilia Gómez-Molero, Pablo Sánchez-Virosta, Henk L. Dekker, Albert de Boer, Elena Eraso, Oliver Bader, Piet W. J. de Groot

**Affiliations:** 1 Albacete Regional Center for Biomedical Research, Castilla—La Mancha Science & Technology Park, University of Castilla-La Mancha, 02008 Albacete, Spain; AnaEsther.Moreno@uclm.es (A.E.M.-M.); emiliagomez803@hotmail.com (E.G.-M.); pablo.s.v@um.es (P.S.-V.); deboer.albert@googlemail.com (A.d.B.); 2 Institute for Medical Microbiology, University Medical Center Göttingen, Kreuzbergring 57, 37075 Göttingen, Germany; 3 Mass Spectrometry of Biomolecules, Swammerdam Institute for Life Sciences, University of Amsterdam, 1098 XH Amsterdam, The Netherlands; h.l.dekker@uva.nl; 4 Department of Immunology, Microbiology and Parasitology, Faculty of Medicine and Nursing, University of the Basque Country (UPV/EHU), 48940 Bilbao, Spain; elena.eraso@ehu.eus

**Keywords:** biofilm formation, adhesion, GPI, cell wall proteins, Als adhesins, *Candida parapsilosis*, candidiasis

## Abstract

*Candida parapsilosis* is among the most frequent causes of candidiasis. Clinical isolates of this species show large variations in colony morphotype, ranging from round and smooth to a variety of non-smooth irregular colony shapes. A non-smooth appearance is related to increased formation of pseudohyphae, higher capacity to form biofilms on abiotic surfaces, and invading agar. Here, we present a comprehensive study of the cell wall proteome of *C. parapsilosis* reference strain CDC317 and seven clinical isolates under planktonic and sessile conditions. This analysis resulted in the identification of 40 wall proteins, most of them homologs of known *Candida albicans* cell wall proteins, such as Gas, Crh, Bgl2, Cht2, Ecm33, Sap, Sod, Plb, Pir, Pga30, Pga59, and adhesin family members. Comparative analysis of exponentially growing and stationary phase planktonic cultures of CDC317 at 30 °C and 37 °C revealed only minor variations. However, comparison of smooth isolates to non-smooth isolates with high biofilm formation capacity showed an increase in abundance and diversity of putative wall adhesins from Als, Iff/Hyr, and Hwp families in the latter. This difference depended more strongly on strain phenotype than on the growth conditions, as it was observed in planktonic as well as biofilm cells. Thus, in the set of isolates analyzed, the high biofilm formation capacity of non-smooth *C. parapsilosis* isolates with elongated cellular phenotypes correlates with the increased surface expression of putative wall adhesins in accordance with their proposed cellular function.

## 1. Introduction

*Candida parapsilosis* is a frequent cause of nosocomial and bloodstream infections, especially in critically ill neonates and immunocompromised patients [1]. Premature infants with a low birth weight are a principal risk group for acquiring *C. parapsilosis* infections due to the requirement of parenteral nutrition using abiotic devices, such as indwelling catheters [2]. This increases the probability of biofilm formation by this yeast, either alone or in combination with other *Candida* species or nosocomial bacteria. Attachment onto and subsequent biofilm formation on medical materials often appears to be the source of *C. parapsilosis* infections in patients in intensive care units or under surgical conditions [3,4,5,6]. Infections with multiple *C. parapsilosis* strains have also been reported [7]. Although biofilm productivity has been reported for many *C. parapsilosis sensu stricto* isolates, data for the related *C. parapsilosis* complex species *Candida orthopsilosis* and *Candida metapsilosis* are less conclusive, and seem to depend on the tested surface material or strain background [8,9].

Biofilms are three-dimensional microbial communities encased in a matrix of extracellular polymeric substance (EPS), which provides protection against the activity of antifungal compounds and the host immune system [10,11]. Biofilms on host tissues are often the result of a complex interplay between multiple microbial inhabitants, such as different *Candida* species and/or commensal bacteria. This may perhaps be different for abiotic surfaces, such as indwelling devices, if a contaminating organism has strong adherence to a particular surface.

*C. parapsilosis* isolates have been shown to produce colonies with different shapes, which roughly can be divided into smooth and various non-smooth morphotypes, such as crepe and concentric. Most isolates present stable dominant phenotypes, but occasional morphotype switching has been observed [12,13]. Furthermore, isolates with irregular non-smooth morphotypes show elongated cellular morphologies (pseudohyphae) and have a higher capacity to form biofilms [12,13].

The *Candida* cell wall is an essential organelle providing cellular strength and forming a protective shield. It also plays a key role in host–pathogen interactions underlying the establishment of fungal infections. These include attachment to host tissues, biofilm formation, invasion, host immune recognition, immune evasion, and proteolytic activities [14,15]. Studies using microscopy have shown that the wall of *Candida* has a bi-layered structure; the inner layer is predominantly composed of a network of polysaccharides (β-glucans and chitin), and is surrounded by a layer of mainly covalently-bound mannoproteins. Known covalently-bound cell wall proteins (CWPs) are either connected to β-1,6-glucans through a glycosylphosphatidylinositol (GPI) anchor remnant or bound to β-1,3-glucan through a mild-alkali sensitive linkage (ASL). Functions of CWPs are manifold, ranging from different families of carbohydrate-active enzymes involved in maturation of cell wall glycans, adhesins, aspartic proteases, heme-iron utilizing proteins, superoxide dismutases, phospholipases, and conserved families of proteins with still unknown or presumably non-enzymatic functions [16]. Several studies have also mentioned wall-associated proteins through disulfide bonds and proteins with known intracellular housekeeping functions, the presence of which at the cell surface was explained to serve moonlighting functions [17].

Attachment of pathogenic *Candida* to abiotic surfaces or host tissues and cell aggregation is mediated by cell wall adhesins. Described in *Candida albicans* are GPI-modified adhesins of the Als, Hwp, and Iff/Hyr protein families [18], and members of all three were found in cell wall preparations [16]. Most intensively studied are the Als proteins, which bind to flexible C-termini of host surface proteins [19], and Hwp1, a hyphal-specific protein that acts as a microbial substrate for epithelial cell transglutaminase to produce cross-links with proteins on mammalian mucosa [20]. In the phylogenetically more distant yeast *Candida glabrata*, these adhesin families are not present; instead, it contains several other families of putative GPI-modified adhesins [18], including well-described Epa lectin-binding terminal galactose residues on host surface proteins [21,22].

Genomic studies have shown that the genome of *C. parapsilosis* encodes a similar repertoire of putative GPI and other CWPs as *C. albicans* and related CTG-clade species, including the same families of adhesins [23]. However, information about which proteins are actually incorporated into the cell wall and are important for adhesion and biofilm formation is limited. A shotgun proteomic study identified up to ten predicted *C. parapsilosis* cell wall proteins in cultures that were induced to form pseudohyphae [24], while phenotypic studies implicated a role for Als7 in adhesion to host extracellular matrix proteins [25] and Rbt1 in biofilm formation [26].

The aim of this study was to provide a comprehensive inventory of covalently-attached cell wall proteins in *C. parapsilosis*, with a special focus on cell wall adhesins. First, the wall proteome of reference strain CDC317, a poor biofilm producer, was characterized under different planktonic conditions. Second, we compared the wall proteomes of smooth and non-smooth isolates with different biofilm-formation capacities. Our results demonstrated a strong increase of putative GPI-modified wall adhesins in isolates with non-smooth morphotypes correlating with their high biofilm productivity, agar invasiveness, and pseudohyphae formation.

## 2. Materials and Methods

### 2.1. Strains, Growth Conditions, and Biofilm Development

*C. parapsilosis* strains used in this study were part of a previously described strain collection [12]. Strains were maintained on YPD (1% yeast extract, 2% peptone, and 2% glucose) or Sabouraud (Oxoid GmbH, Wesel, Germany) agar. Colony morphology was studied on YPD agar with or without 5 mg/mL of Phloxine B [13] (Sigma, Steinheim, Germany) and on cornmeal agar [27] (Becton Dickinson and Company, Le-Pont de-Claix, France). Inocula for liquid culturing were prepared by pre-culturing overnight in YPD, unless stated otherwise. Liquid cultures for proteomic studies were grown at 30 or 37 °C in YPD and harvested at the exponential phase (optical density (OD)_600_ = 1–2) or after 24 h of growth (stationary phase) by centrifugation. For the development of biofilms on polystyrene, exponentially growing cultures in YPD were adjusted to OD_600_ = 1 with fresh YPD. Twenty mL of the cell suspensions were seeded into sterile petri dishes and incubated for 24 h at 37 °C in a moist environment. After incubation, unbound cells were removed, and the biofilms were gently rinsed with mQ water. Bound cells were collected by scraping. A similar procedure was used for the development of biofilms onto silicone elastomer. In this case, sterile 25 cm^2^ non-reinforced silicone sheets (AMT Aromando Medizintechnik GmbH, Düsseldorf, Germany) were deposited into petri dishes containing sterile glass beads to prevent direct contact between the silicone and plastic. After incubation, the sheets were rinsed and transferred to a new petri dish, and biofilm cells were collected by scraping. For all proteomic samples, independent duplicate cultures were obtained and analyzed separately.

### 2.2. Biofilm Formation onto Polystyrene and Silicone

Biofilm production of *C. parapsilosis* strains to plastic (polystyrene) was determined in microtiter plates with the crystal violet (CV, Sigma) assay after 24 h of incubation in YPD at 37 °C in a moist environment, as detailed in [12].

For the determination of biofilm formation capacity onto silicone elastomers, overnight pre-cultures were adjusted to a cell density of 0.8 McFarland, and 100 μL were added to 15 mL glass tubes containing 4 mL of YPD and a 1 cm^2^ silicone square. Cells and silicone pieces were incubated overnight at 37 °C and 200 rpm. Silicone pieces were then removed and deposited in a 12-well plate (Greiner Bio-One, Frickenhauser, Germany), and unbound cells were washed off with phosphate-buffered saline (PBS). Washed silicone pieces were transferred to wells of a new plate containing 1 mL of fresh PBS, biofilms were collected by scraping the silicone surface, and cells were quantified by measuring the OD_595_ of a 1:100 dilution in PBS using an MRX-TC Revelation microplate reader (Dynex Technologies GmbH, Denkendorf Germany).

### 2.3. Antifungal Susceptibility

Antifungal drug susceptibility was determined following EUCAST EDef 7.2 standards [28]. Amphotericin B (AMB), fluconazole (FLZ), posaconazole (POS), and voriconazole (VRZ) were obtained from Discovery Fine Chemicals Ltd. (Bournemouth, UK), caspofungin (CAS) from Merck Sharp & Dohme Corp (MSD, Haar, Germany), and micafungin (MFG) from Astellas. Minimum inhibitory concentration (MIC) values were determined after 24 h at 37 °C.

### 2.4. Agar Invasion Capacity

Agar invasion capacity of *C. parapsilosis* strains was tested as previously described by [12,13]. Cells were plated on YPD agar supplemented with 5 mg/mL of Phloxine B and allowed to grow for ten days. Agar invasion was scored by gently scraping colonies with an inoculation loop while washing off the cells under running water at day ten of incubation. Agar invasion was defined on a rating scale from low (1) to high (5) invasion as described [12].

### 2.5. Microscopy

Colonies or biofilms in microplates prior to CV staining were observed through a Zeiss Axiovert 200M microscope (Carl Zeiss AG, Oberkochen, Germany) at 10× and 40× magnification. Cell morphology of overnight cultures in YPD was monitored by phase-contrast microscopy. For the latter, cells were stained for 20 min with 0.1% Blankophor P solution, washed with PBS, and fixed with 100% methanol for 5 min. After washing with PBS, cells were embedded in Mowiol 4-88 (Sigma) and observed at 100× magnification.

### 2.6. Cell Wall Isolation

*C. parapsilosis* cell walls were purified following a previously described protocol [29,30,31]. Briefly, cells were disrupted with 0.4–0.6 mm glass beads (Sartorius, Goettingen, Germany) in a Fastprep-24 machine (MP Biomedicals, Eschwege, Germany) during at least six runs of 30 s at 6.5 m/s. Complete cell breakage was checked with a light microscope and, if needed, additional runs were performed. After cell breakage, cell wall material was washed extensively with 1 M of NaCl (each washing step in the protocol being followed by centrifugation to pellet the cell wall material) and incubated twice for 10 min with sodium dodecyl sulfate (SDS) extraction buffer (50 mM of Tris HCl, 2% SDS, 100 mM of Na-EDTA, 150 mM of NaCl, 0.8% β-mercaptoethanol, pH = 7.8) in a boiling water bath to remove any non-covalently bound proteins. Finally, the walls were washed extensively with mQ water, freeze-dried, and stored at −20 °C until use.

### 2.7. Mass Spectrometric Analysis

Reduction, *S*-alkylation, and proteolytic digestion of cell walls with Trypsin Gold (Promega, Madrid, Spain) were performed as described [32]. Released peptides were freeze-dried and taken up in 50% acetonitrile (ACN) and 2% formic acid. Peptide concentrations were estimated by measuring the OD_214_ and calibrating against a Peptide Calibration Mixture (Thermo Scientific, Landsmeer, The Netherlands). Samples were diluted with a 0.1% trifluoroacetic acid (TFA) solution to reach a peptide concentration of about 250–500 fmol μL^−1^. Samples were analyzed using an amaZon Speed Iontrap with a CaptiveSpray ion source (Bruker, Leiderdorp, The Netherlands) coupled to an EASY-nLC II (Proxeon, Thermo Scientific) chromatographic system. Peptide samples were injected and separated with an eluent flow of 300 nL min^−1^ on an EASY Column of 10 cm (analytical column SC200 coupled to a 2 cm trap column SC001 pre-column (Thermo Scientific)) using a 50 min gradient of 0–50% ACN and 0.1% formic acid. Peptide precursor ions above a predefined threshold ion count were selected for low-energy, collision-induced dissociation (CID) to obtain fragmentation spectra of the peptides. The amount of sample used per run was 0.5–1.0 pmol of peptide material.

### 2.8. MS/MS Database Searching

Raw MS/MS data were processed with Data Analysis software (Bruker, Billerica, MA, USA). Resulting.mgf data files were used for searching with licensed Mascot software (Version 2.5.1) against a non-redundant *C. parapsilosis* protein database prepared with CDC317 protein sequences downloaded from NCBI. Simultaneously, searches were performed against a common contaminants database (compiled by the Max Planck Institute of Biochemistry, Martinsried, Germany) to minimize false identifications. Mascot search parameters were: a fixed modification of carbamidomethylated cysteine, variable modification of oxidized methionine, trypsin with the allowance of one missed cleavage, peptide charge state +1, +2, and +3, and decoy database activated. Peptide and MS/MS mass error tolerances were 0.3 and 0.6 Da, respectively. Probability-based MASCOT scores (http://www.matrixscience.com/, accessed on 15 February 2021) were used to evaluate the protein identifications with a 1% false discovery rate as the output threshold. Peptides with scores lower than 20 were ignored. Unmatched peptides were subjected to a second Mascot search with semitrypsin as the protease setting, N/Q deamidation as the extra variable modification, and a cutoff peptide ionscore of 40. Semitryptic peptides identified in the second search served solely to extend the sequence coverage of proteins identified in the first trypsin search. Protein identifications based on a single peptide match were only taken into consideration if identified in multiples and at least one time in duplicate samples. The validity of single peptide matches was further verified by manual inspection of MS/MS spectra in the raw data using the Data Analysis software. GPI protein predictions were performed as described [33]. The total number of peptides (TP) identified for each protein was determined by adding up all MS/MS fragmentation spectra, leading to protein identification in the two duplicate samples.

## 3. Results

### 3.1. The Cell Wall Proteome of Reference Strain CDC317 under Planktonic Conditions

The first aim of this study was to obtain a comprehensive inventory of covalently-bound cell wall proteins in the widely used *C. parapsilosis* reference strain CDC317. As this strain is not a producer of thick biofilms, we applied planktonic conditions and compared exponentially growing (OD_600_ = 1–2) and stationary phase (after 24 h of growth) cultures in YPD, both at 30 °C and 37 °C. Our cell wall purification protocol included stringent washing steps, removing non-covalently-bound proteins. The so-called cell-wall shaving approach with trypsin was employed to release peptides from the purified wall matrices, which subsequently were analyzed by tandem mass spectrometry (LC-MS/MS). Results obtained with the reference strain are presented in Table 1; for mass spectrometric details of individual peptides, see Supplementary Table S1. Each of the four conditions yielded between 20 and 26 protein identifications, and a total of 27 wall proteins were identified in CDC317. A majority of 18 proteins (Ywp1, Phr1, Phr2, Pga4, MP65, Bgl2, Crh11, Utr2, Cht2, Plb5, Plb51, Rbt5, Ecm33, Pir1, Pga30, Pga59, Tos1, and Ssr1) were identified in all four conditions, indicating little variation between the wall proteomes in these planktonic cultures and good consistency of our data. A smaller set (Als6, Rbt1, Sod4, Sap9, Sap91, Ecm331, Sun41, Rhd3, and Pga1) was not identified in all four conditions. Judged from the total and different number of peptides identified for each protein (Table 1), these wall proteins generally seemed to be less abundant. Thus, rather than regulatory issues, the reason for their absence in any sample may be that the level of most of these proteins in the wall is close to the lower detection limit of our instrumentation. Ecm331 and Sap91 may be an exception to this. Sap91 was identified only—but with four different peptides—in cells grown to the stationary phase at 37 °C. In this condition, Ecm331 showed higher peptide counts and sequence coverage than in other conditions. Identified peptide counts further suggested a higher abundance of Phr2, Ecm33, and Ssr1 in walls at 37 °C, especially at the stationary phase. Consistent with this, all five proteins were also observed in all clinical isolates grown under the same condition, as well as in biofilms (Table 2 and Table 3).

### 3.2. Selection and Characteristics of C. parapsilosis Isolates with Different Biofilm Formation Status

Only a few peptides from proteins belonging to Als and Hwp adhesin families were identified in the low biofilm-forming (LBF) reference strain CDC317, while peptides from Iff/Hyr proteins were absent (Table 1). In *C. glabrata*, the incorporation of putative adhesins was shown to depend on the adhesion/biofilm formation capacity of clinical isolates [31]. This prompted us to investigate if this was also the case in *C. parapsilosis*. Therefore, the second aim of this study was to compare the wall proteomes of strains with different capacities to form biofilms.

**Figure 1:**
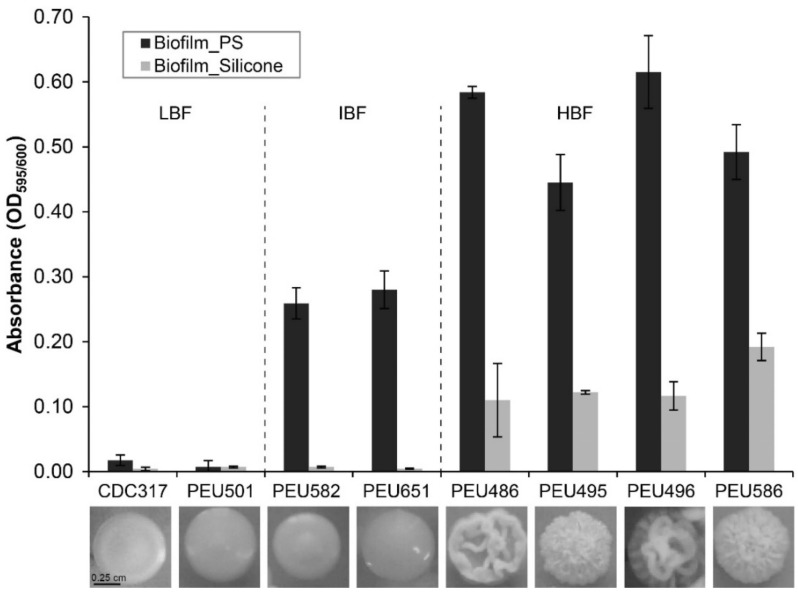
Biofilm formation onto polystyrene and silicone. The quantity of cell material that adhered to polystyrene (PS) or silicone after 24 h of incubation in YPD at 37 °C was determined as detailed in the Materials and Methods section. Data shown are averages ± SDs of at least two independent biological experiments with four technical replicates each. Photographs below show the dominant colony morphotype of each strain after 96 h of growth on YPD agar at 37 °C.

**Figure 2:**
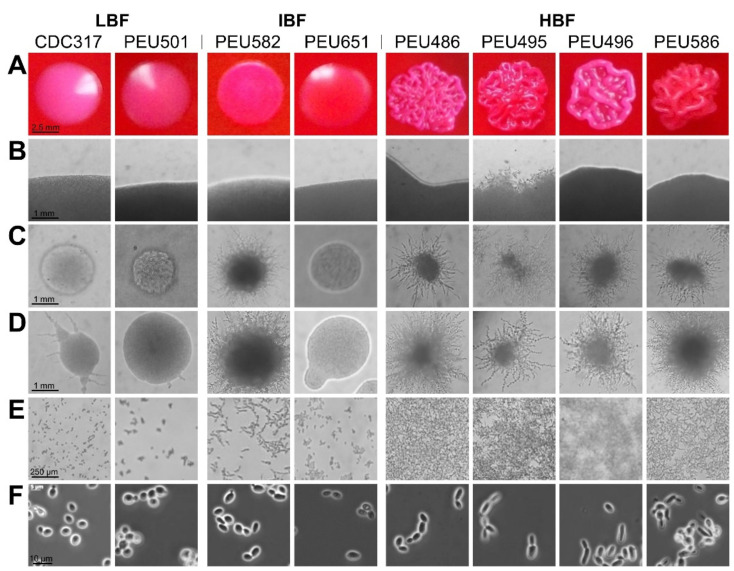
Colony morphotype and cellular morphology of LBF, IBF, and HBF strains. (**A**) Predominant colony morphotypes on YPD + phloxine B agar after 96 h of growth. (**B**) Borders of colonies grown on YPD agar for 96 h. (**C**,**D**) Colonies grown on cornmeal agar for 48 h (**C**) and 96 h (**D**). (**E**) Cells/biofilms adhered to polystyrene after 24 h of incubation. (**F**) Cellular morphology after overnight culturing in liquid YPD observed by phase-contrast microscopy.

Seven *C. parapsilosis* clinical isolates with either low (LBF; OD_595_ < 0.08), intermediate (IBF; 0.2–0.3), or high (HBF; > 0.4) biofilm formation capacity on polystyrene and representative and stable morphotypes were selected from a previously described collection [12]. Among these selected strains, the four HBF isolates were the only strains that also formed considerable biofilms on silicone elastomers, PEU586 showing the highest level (Figure 1). The dominant morphotype of HBF strains was non-smooth crepe versus smooth for the selected LBF and IBF strains (Table 2 and Figure 2). The HBF strains also showed higher agar invasion and more frequent appearance of elongated pseudohyphae compared to the LBF and IBF isolates. In contrast, no obvious correlation between biofilm formation status and antifungal drug susceptibility profiles or clinical origin of these isolates was detected (Table 2), in line with the data for the whole strain collection [12]

Proteomic analysis of IBF and HBF strains was performed with walls isolated from 24 h biofilms on polystyrene (Table 3). The growth of a sufficient biofilm for wall isolation and proteomic analysis on silicone was more difficult, therefore, this was performed only with the strongest silicone biofilm-producing strain, PEU586. LBF strain PEU501, like CDC317, hardly developed biofilms on any surface. For comparative reasons, all strains were therefore also analyzed under planktonic conditions by culturing to the stationary phase at 37 °C.

### 3.3. The Core Cell Wall Proteome of C. parapsilosis

Analysis of the proteomic data of the seven *C. parapsilosis* clinical isolates confirmed and completed the image of the wall proteome obtained by analysis of CDC317. All 27 proteins identified in strain CDC317 were also identified in at least two of the clinical isolates, and a total of 40 proteins were identified (Table 3, see Supplementary Table S1 for mass spectrometric details of individual peptides). Of the 18 proteins that were identified in all four CDC317 culturing conditions, only Pga59 was not identified in all seven clinical isolates. These ubiquitous proteins therefore can be considered a part of the core cell wall proteome of *C. parapsilosis*.

All 40 identified proteins were classical secretory proteins with N-terminal signal peptides, and 37 also carried C-terminal GPI-anchoring signals or, by analogy to *C. albicans*, belonged to families of ASL wall proteins [34]. Thirty-four proteins were orthologs or closest homologs of proteins that have also been identified in wall proteomic studies of *C. albicans* [16]. Of the remaining six proteins, three were predicted GPI proteins: Pga1 and Pga53 were orthologs of *C. albicans* GPI proteins with unknown function, and CPAR2_701390 also was a protein with unknown function, for which NCBI Blast did not reveal homologs in other *Candida* spp., except for an ortholog in the closely related *C. orthopsilosis*. CPAR2_701390 showed 32% sequence identity with adjacent ORF CPAR2_701380, another predicted GPI protein. Interestingly, these two proteins shared a conserved pattern of eight cysteine residues in their N-terminal parts reminiscent of, but not identical to, CFEM domains [35] that are present in Rbt5 family proteins and Ssr1. The last three identified proteins, Nce102, CPAR2_805040, and CPAR2_403880, were secretory proteins of unknown function lacking predicted GPI anchoring signals, which made their presence among covalently-bound proteins surprising. For all three proteins, their identification was based on a single peptide match that was found in planktonic as well as biofilm samples of only one or two of the clinical isolates. Other proteins identified in clinical isolates but not in CDC317 were the putative adhesins Als7, Als11, and Hyr31, the Cht2 chitinase homologs Cht21 and Cht22, the aspartic proteases Sap10 and Sap92, and the Rbt5 family protein Csa1, Pga53, and CPAR2_701390. Of these, Hyr31, Cht21, Cht22, Csa1, and Nce102 were identified in HBF strains only. On the contrary, Rhd3 was the only protein that was not identified in any of the HBF strains.

Comparison of cell wall proteomes in biofilms versus planktonic conditions yielded only some minor proteomic differences (Table 3). Hyr31 and Cht22 were detected only in biofilm samples, while Pga1 was detected only in planktonic conditions. Similar to the above explanation for possible differences among CDC317 samples, a generally low abundance of these three proteins (judged from peptide counts, Supplementary Table S2) with rather differential regulation may perhaps explain their identification in only few samples.

### 3.4. Incorporation of Wall Adhesins Is Increased in C. parapsilosis Isolates with High Biofilm-Formation Capacity

When the proteomic data of the different clinical isolates were analyzed in a semi-quantitative manner by peptide counting (Table 3 and Supplementary Table S2), a very notable difference was observed between HBF isolates and LBF + IBF isolates in the number of different (DP) and total (TP) peptides identified for members of Als, Hwp, and Iff/Hyr putative adhesin families. In 37 °C stationary phase planktonic samples of LBF and IBF strains, the DP for such proteins varied from 3–10, and the TP from 33–117. In the two IBF biofilm samples, the DP for putative adhesins was 3–14, with 212–213 TP identified. However, in HBF strains, the number of putative adhesin peptides was much higher: 39–51 DP and 433–592 TP in 37 °C stationary phase planktonic samples, and 43–50 DP and 484–698 TP in biofilm samples. With the calculated average DP and TP for both groups of strains, one can infer that, in the HBF strains, putative adhesins were 8.1 (DP) to 6.4 (TP) times more abundant in planktonic and 5.6 (DP) to 9.6 (TP) times more abundant in biofilm cultures compared to LBF/IBF strains. In contrast to this higher abundance of putative adhesin peptides in HBF strains, the average numbers of all identified wall peptides were almost identical in the two groups, with HBF/(LBF + IBF) ratios of 1.08 for average DP and 1.03 for average TP. Thus, our analysis clearly showed upregulation of putative adhesins in HBF strains, which seems to be five- to tenfold higher under both planktonic and biofilm conditions.

A total of six different putative adhesins were identified in the walls of HBF strains: in addition to Als6, Rbt1, and Ywp1, which were also found in CDC317, Als proteins Als7 and Als11 and Iff/Hyr family protein Hyr31 were identified. Based on DP and TP counts, the two Als proteins appeared strongly upregulated in HBF strains, and this was also the case for Rbt1. Hyr31 was only detected in HBF strains, thus, its expression may also be related to biofilm formation capacity; however, the low peptide coverage of this protein did not allow hard conclusions, as commented above. Ywp1, on the other hand, appeared to be most abundant in the two LBF strains PEU501 and CDC317 (under all four planktonic conditions analyzed). This was remarkable for a protein from an adhesin family, as discussed below.

Finally, the wall proteome of PEU586 biofilms grown on silicone was almost identical to biofilms on polystyrene and planktonic stationary phase cultures, and no differential incorporation of putative adhesins was observed. Surprisingly, adhesin Als6 was absent in all samples of this HBF strain.

## 4. Discussion

Here, we have presented a comprehensive study of the covalently-bound cell wall proteome in *C. parapsilosis*. Our study compiles data from eight different strains with different capacities to form biofilms. Analyzed culturing conditions include planktonic exponentially growing and stationary phase cultures at both 30 and 37 degrees, as well as biofilm cultures on polystyrene and silicone. The retention of non-covalently bound or moonlighting proteins was avoided by executing stringent washing steps during the cell wall isolation procedure. In the past, we have applied this procedure successfully to various other fungal species, including the related yeasts *C. albicans*, *C. glabrata*, and *Saccharomyces cerevisiae*, as well as the filamentous *Aspergillus nidulans* [29,31,32,36]. The analyzed *C. parapsilosis* samples yielded a total of 40 identifiable wall proteins, including all genuine wall proteins identified by Karkowska-Kuleta and colleagues [24].

Sequences of all identified proteins contain canonical signal peptides for secretion, and are thus destined to reach the cell surface. Most are homologs of *C. albicans* proteins that have previously been identified in cell wall preparations and/or are predicted GPI proteins. The only three exceptions to this are Nce102, CPAR2_805040, and CPAR2_403880. All different classes or families of wall proteins known from *C. albicans* are represented in our list, including Als, Hwp, and Iff/Hyr putative adhesins, Gas/Phr, Bgl2, Crh, and Cht2 glycoside hydrolyases with diverse functions in modification or crosslinking of cell wall polysaccharides during growth and division, aspartic proteases with proposed roles in the shedding of wall proteins and host tissue invasion, phospholipases, Ecm33, Rbt5, Pga30, Pga59, and Sun41 family proteins, superoxide dismutase Sod4, and a Pir protein with a proposed non-enzymatic role in β-1,3-glucan crosslinking [16].

Explaining why Nce102, CPAR2_805040, and CPAR2_403880 are encountered among covalently-bound wall proteins is challenging. It may be that they belong to the class of alkali-extractable ASL proteins. For the best-studied ASL wall protein in *S. cerevisiae*, Cis3/Pir4, it has been shown that a conserved glutamine-rich repeat sequence (Pir repeat) is essential for forming a covalent link to cell wall β-1,3-glucan [37]. However, various other ASL proteins or ASL protein homologs in *S. cerevisiae*, *C. albicans*, and *C. glabrata* do not contain such Pir repeats, and the nature of their alkali-sensitive linkages remains poorly understood to date. Another possibility is that the three proteins are strongly associated to the cell wall matrix in a different way, perhaps even non-covalently. The three proteins were mass spectrometrically identified by a single peptide and only in one or two clinical isolates, indicating a low overall abundance in our samples. It is noteworthy that the *C. albicans* orthologs of Nce102 and CPAR2_805040 have been reported to be upregulated in biofilms [38,39], and the latter has putative adhesin properties, according to the FungalRV predictor [40]. We did not attempt protein extractions with mild alkali to investigate the issue in more detail, as we consider this beyond the scope of this wall proteomic shotgun study.

Various studies have reported considerable dynamics of the wall proteome in *C. albicans* when altering growth conditions, such as pH [41], carbon source [42], the presence of fluconazole in the growth medium [43], or conditions that induce hyphal formation [44]. The latter triggers specific expression of various wall proteins, for instance the adhesins Als3, Hwp1, and Hyr1, and the superoxide dismutase Sod5, while some other proteins (Rhd3, Sod4, and Ywp1) showed decreased levels in the walls of hyphae. Our study in *C. parapsilosis* did not show major intra-strain, growth condition-dependent, wall protein dynamics, neither when comparing different planktonic growth conditions for CDC317 (exponential growth versus stationary phase at 30 °C and 37 °C) nor when comparing planktonic cultures and biofilms for six IBF and HBF clinical isolates. In contrast, clear changes in the wall proteome of *C. parapsilosis* were observed depending on the biofilm-forming capacity of the different clinical isolates analyzed, as discussed below. We propose that the constitutively observed proteins, that is, the ones that were detected in most isolates and under most conditions and that generally yielded the highest peptide counts, constitute the core cell wall proteome of *C. parapsilosis*.

The pathogenicity of *C. parapsilosis* is related to its ability to adhere to host tissues and to form a biofilm on medical devices governed by proteins with adhesive functions [5]. *C. parapsilosis* contains the same families of putative adhesins as *C. albicans* and other CTG-clade *Candida* species [23]. Comparison of adhesive capacity to buccal epithelial cells (BECs) or acrylic surfaces of various *C. albicans* and *C. parapsilosis* clinical isolates did not reveal significant differences between the two organisms [45]. In the same study, higher adhesion to BECs was reported for superficial isolates than for systemic isolates of *C. parapsilosis* [45]. In our strain collection, no relation between biofilm-forming capacity and the host site of infection was detected nor any relation between biofilm-forming capacity and antifungal drug susceptibility [31]. However, in accordance with earlier studies, HBF phenotypes in our collection correlate with the appearance of non-smooth colonies, increased pseudohyphae formation, and high agar invasion capacity [12,13,46,47]; this is also the case for the HBF representatives used in this study that originated from ear and nose swabs (2x), skin, and urine samples.

Similar to our earlier studies with hyperadhesive strains in *C. glabrata* [31], the four HBF isolates showed increased incorporation of putative adhesins compared to LBF and IBF isolates. While few peptides from putative adhesins of Als, Hwp, or Iff/Hyr families were identified in LBF and IBF strains, a clear five- to tenfold increase in the number of peptides originating from these protein families was detected in HBF strains. *C. glabrata* is phylogenetically more distant to *C. parapsilosis* and *C. albicans*, and does not contain Als, Hwp, or Iff/Hyr families. Instead, it contains different families of adhesins, for instance, Epa proteins that act as lectins to make connections with host epithelia [21,22], as well as many other putative GPI-modified adhesins, the functions of which are still uncovered [48]. 

Two recent studies reported on *C. parapsilosis ALS* gene expression using real-time PCR [49] or RNAseq analysis [50] of strains with different adhesive capacity under adhesion-inducing as well as non-inducing conditions. Both studies suggest that, despite some observed upregulation of *ALS7*/CPAR2_404800 in one strain under adhesion-inducing conditions [50], the regulation of *ALS* genes depended more on phenotypic strain variability than on growth conditions. Our data concur with this view, and indicate that this is also the case for the Hwp and Hyr adhesin families. However, detailed analysis of genomic variations that may lead to altered adhesin expression will have to await the availability of sequence information of isolates with different biofilm formation capacities. Als6/CPAR2_404790 has been reported as the most highly expressed Als protein [49], but, strikingly, this protein was absent in the walls of HBF strain PEU586. Therefore, the high adhesive capacity of this strain to silicone elastomer does not seem to be related to Als6 expression.

The levels of Als7, Als11, and Rbt1 were especially highly upregulated in HBF strains. This also seems to be the case for Hyr31, although most of its identified peptides are non-unique and shared by multiple Iff/Hyr family members. For Als7 and Rbt1, our data concur with phenotypic studies that demonstrated their involvement in adhesion to ECM proteins [25] and biofilm formation [26], respectively. Ywp1 presents a special case. Its ortholog in *C. albicans* has been categorized as a putative Hwp-like adhesion, because it contains repeat motifs that are characteristic for this protein family [18]. However, the protein is decreased in hyphae, unlike several adhesins, and *C. albicans* mutants deficient in Ywp1 showed increased adhesiveness and biofilm formation. Therefore, rather than acting as an adhesin, Ywp1 was proposed to have anti-adhesive properties and promote the dispersal of yeast cells [51]. This view is consistent with our data, showing that Ywp1 was most abundant in the two LBF strains.

In conclusion, this study provides a detailed inventory of covalently-associated wall proteins in the pathogenic yeast *C. parapsilosis*. The *C. parapsilosis* wall proteome comprises a stable set of core proteins, including all functions that are required for proper wall synthesis and functioning, supplemented by proteins with putative adhesive functions. Non-smooth HBF strains show increased incorporation of putative adhesins, correlating with the higher capacity of these strains to adhere, form biofilm, develop pseudohyphae, and invade agar.

## Figures and Tables

**Table 1:** MS/MS identification of cell wall proteins in *C. parapsilosis* CDC317.

CGD Name (Proposed Name)	Ortholog (o) or Closest Homolog (h) in *C. albicans*	Functional Class or Family	Size (aa)	Exponential Growth		Stationary Phase	
				30 °C	37 °C	30 °C	37 °C
				Identified Peptides DP/TP ^a^			
Putative adhesins							
CPAR2_404790 (Als6)	Als6/C3_06190C (o)	Als family	2392	1/2	1/1		
Rbt1/CPAR2_403510	Rbt1/C4_03520C (o)	Hwp family	812				1/2
Ywp1/CPAR2_806670	Ywp1/C2_08590W (o)	Hwp family	522	1/86	1/73	1/72	2/100
Carbohydrate active enzymes							
CPAR2_302140 (Phr1)	Phr1/C4_04530C (o)	CaZy GH72 Gas/Phr family	532	7/9	10/13	3/3	3/3
CPAR2_109660 (Phr2)	Phr2/C1_00220W (o)	CaZy GH72 Gas/Phr family	566	14/99	16/141	12/90	19/189
CPAR2_100110 (Pga4)	Pga4/C5_05390C (o)	CaZy GH72 Gas/Phr family	464	12/92	13/91	12/84	13/108
CPAR2_407410 (MP65)	MP65/C2_10030C (o)	CaZy GH17 Bgl2 family	372	12/75	12/96	9/45	12/77
CPAR2_401600 (Bgl2)	Bgl2/C4_02250C (o)	CaZy GH17 Bgl2 family	308	4/12	6/10	3/3	6/12
CPAR2_400860 (Crh11)	Crh11/C4_02900C (o)	CaZy GH16 Crh family	490	10/215	10/195	7/127	9/263
CPAR2_503190 (Utr2)	Utr2/C3_01730C (o)	CaZy GH16 Crh family	461	4/24	3/31	4/16	3/31
CPAR2_502140 (Cht2)	Cht2/C5_04130C (o)	CaZy GH18 Chitinase	584	28/604	34/506	24/393	24/326
Other enzymes							
CPAR2_213080 (Sod4)	Sod4/C2_00660C (o)	Superoxide dismutase	215	1/1	1/2		1/3
CPAR2_102610 (Sap9)	Sap9/C3_03870C (o)	Aspartic protease	597	1/1	1/2		1/1
CPAR2_702730 (Sap91)	Sap9/C3_03870C (h)	Aspartic protease	528				4/7
CPAR2_804680 (Plb5)	Plb5/C1_08230C (o)	Phospholipase	724	5/10	6/19	6/12	7/37
CPAR2_808920 (Plb51)	Plb5/C1_08230C (h)	Phospholipase	884	5/23	8/27	6/27	11/70
Non-enzymatic or unknown function							
CPAR2_402910 (Rbt51)	Rbt5/C4_00130W (h)	Iron acquisition Rbt5 family	232	1/24	1/25	1/3	1/45
CPAR2_108560 (Ecm33)	Ecm33/C1_03190C (o)	Ecm33 family	433	14/112	18/201	11/115	18/302
Ecm331/CPAR2_100710	Ecm331/C5_02460C (o)	Ecm33 family	438		1/1	1/2	3/8
CPAR2_603090 (Sun41)	Sun41/C6_00820W (o)	Sun family	431				1/2
CPAR2_806490 (Pir1)	Pir1/C2_08870C (o)	Pir family	400	9/149	16/203	13/282	20/547
Pga30/CPAR2_402000	Pga30/C4_04070C (o)	Pga30 family	285	5/9	2/8	11/60	8/19
Rhd3/CPAR2_402010	Rhd3/C4_04050C (o)	Pga30 family	273	1/2			1/1
CPAR2_603340 (Pga59)	Pga59/C4_02370C (o)	Pga59/Pga62 family	138	1/9	1/12	1/5	1/8
CPAR2_503650 (Tos1)	Tos1/C3_01550C (o)	Unknown function	444	2/5	3/9	2/3	3/6
CPAR2_301540 (Ssr1)	Ssr1/C7_00860W (o)	Unknown function	241	11/133	13/213	10/144	14/258
CPAR2_200370 (Pga1)	Pga1/CR_10480W (o)	Unknown function	129		1/1	1/1	1/2

^a^ DP, number of different peptides identified; TP, total number of peptides identified.

**Table 2:** Characteristics of strains used in this study.

Strain (Origin)	Dominant Morphotype (Sporadic)	Biofilm Formation	Invasiveness ^b^	Cell Shape ^c^	MIC (µg/mL) ^d^					
					AMB	FLZ	POS	VRZ	CAS	MFG
CDC317 (Ref. strain, skin)	Smooth	LBF ^a^	1	Yeast	0.125	4–16	0.125–0.25	0.125–0.25	1	1–2
PEU501 (Ear-nose swab)	Smooth	LBF	1	Yeast	0.125–0.25	0.25–0.5	0.063–0.125	0.031–0.063	0.25	1
PEU582 (Urine)	Smooth (Crepe)	IBF	2	Yeast	0.125	0.5–1	0.063–0.125	0.031	0.5–1	2
PEU651 (Indwelling device)	Smooth	IBF	2	Yeast	0.125–0.25	4	0.063–0.125	0.031–0.063	0.5–1	0.125–0.25
PEU486 (Skin)	Crepe (Smooth/concentric)	HBF	5	Yeast & PH	0.125	1	0.031–0.063	0.031–0.063	1	1–2
PEU495 (Urine)	Crepe (Smooth)	HBF	5	Yeast & PH	0.125	0.5–1	0.031–0.063	0.125–0.25	0.25–0.5	1–2
PEU496 (Ear-nose swab)	Crepe (Smooth/crater)	HBF	5	Yeast & PH	0.125	0.5	0.063	0.031	2	2
PEU586 (Ear-nose swab)	Crepe (Smooth/concentric)	HBF	5	Yeast & PH	0.125	1	0.125	0.031	2	2

^a^ LBF, IBF, and HBF, low, intermediate, and high biofilm formation capacity, respectively. ^b^ Invasiveness ranges from low (1) to high (5). ^c^ Cellular morphology after overnight culturing in yeast extract, peptone, dextrose (YPD), PH, pseudophyphae. ^d^ Minimal inhibitory concentrations (MICs) of amphotericin B (AMB), fluconazole (FLZ), posaconazole (POS), voriconazole (VRZ), caspofungin (CAS), and micafungin (MFG).

**Table 3:** Identified proteins in cell walls from *C. parapsilosis* clinical isolates.

Identified Protein (Proposed Name)	*C. albicans* Ortholog (o) or Homolog (h)	Functional Class or Family	Characteristics	LBF	IBF		HBF			
				PEU501	PEU582	PEU651	PEU486	PEU495	PEU496	PEU586
				St ^a^	St/B_PS	St/B_PS	St/B_PS	St/B_PS	St/B_PS	St/B_PS/B_S
Putative adhesins				117 ^b^	68/90	33/27	564/525	433/484	592/582	466/698/509
CPAR2_404780 (Als11)	Als1/C6_03700W (h)	Als family	SP, GPI	+ ^c^	+/+	−/−	+/+	+/+	+/+	+/+/+
CPAR2_404790 (Als6)	Als6/C3_06190C (o)	Als family	SP, GPI	−	+/+	−/−	+/+	+/+	+/+	−/−/−
Als7/CPAR2_404800	Als7/C3_06320W (o)	Als family	SP, GPI	+	−/−	−/−	+/+	+/+	+/+	+/+/+
CPAR2_600430 (Hyr31)	Hyr3/C5_00730W (h)	Iff/Hyr family	SP, GPI	−	−/−	−/−	−/+	−/−	−/+	−/−/−
Rbt1/CPAR2_403510	Rbt1/C4_03520C (o)	Hwp family	SP, GPI	+	+/+	+/+	+/+	+/+	+/+	+/+/+
Ywp1/CPAR2_806670	Ywp1/C2_08590W (o)	Hwp family	SP, GPI	+	+/+	+/+	+/+	+/+	+/+	+/+/+
Non-adhesin proteins—core proteome				2206	2160/2000	2605/2261	1871/1550	1661/2139	1915/1693	1818/2046/2097
Carbohydrate active enzymes										
CPAR2_302140 (Phr1)	Phr1/C4_04530C (o)	Gas/Phr family	SP, GPI ^d^	+	+/+	+/+	+/+	+/+	+/+	+/+/+
CPAR2_109660 (Phr2)	Phr2/C1_00220W (o)	Gas/Phr family	SP, GPI	+	+/+	+/+	+/+	+/+	+/+	+/+/+
CPAR2_100110 (Pga4)	Pga4/C5_05390C (o)	Gas/Phr family	SP, GPI	+	+/+	+/+	+/+	+/+	+/+	+/+/+
CPAR2_407410 (MP65)	MP65/C2_10030C (o)	Bgl2 family	SP, ASL	+	+/+	+/+	+/+	+/+	+/+	+/+/+
CPAR2_401600 (Bgl2)	Bgl2/C4_02250C (o)	Bgl2 family	SP, ASL	+	+/+	+/+	+/+	+/+	+/+	+/+/+
CPAR2_400860 (Crh11)	Crh11/C4_02900C (o)	Crh family	SP, GPI	+	+/+	+/+	+/+	+/+	+/+	+/+/+
CPAR2_503190 (Utr2)	Utr2/C3_01730C (o)	Crh family	SP, GPI	+	+/+	+/+	+/+	+/+	+/+	+/+/+
CPAR2_502140 (Cht2)	Cht2/C5_04130C (o)	Chitinase	SP, GPI	+	+/+	+/+	+/+	+/+	+/+	+/+/+
CPAR2_502130 (Cht21)	Cht2/C5_04130C (h)	Chitinase	SP, GPI	−	−/−	−/−	+/+	+/+	−/−	+/+/+
CPAR2_502120 (Cht22)	Cht2/C5_04130C (h)	Chitinase	SP, GPI	−	−/−	−/−	−/+	−/+	−/−	−/−/+
Other enzymes										
CPAR2_213080 (Sod4)	Sod4/C2_00660C (o)	Superoxide dismutase	SP, GPI	+	−/−	−/+	+/+	−/+	+/+	−/−/−
CPAR2_102610 (Sap9)	Sap9/C3_03870C (o)	Aspartic protease	SP, GPI	+	−/+	+/+	−/+	−/+	+/+	+/+/+
CPAR2_702730 (Sap91)	Sap9/C3_03870C (h)	Aspartic protease	SP, GPI	+	+/+	+/+	+/+	+/+	+/+	+/+/+
CPAR2_702720 (Sap92)	Sap9/C3_03870C (h)	Aspartic protease	SP, GPI	−	+/+	+/+	+/+	−/−	−/−	−/−/−
CPAR2_500920 (Sap10)	Sap10/C4_04470W (o)	Aspartic protease	SP, GPI	−	−/+	+/+	−/+	−/−	−/−	−/−/−
CPAR2_804680 (Plb5)	Plb5/C1_08230C (o)	Phospholipase	SP, GPI	+	+/+	+/+	+/+	+/+	+/+	+/+/+
CPAR2_808920 (Plb51)	Plb5/C1_08230C (h)	Phospholipase	SP, GPI	+	+/+	+/+	+/+	+/+	+/+	+/+/+
Non-enzymatic or Unknown function										
CPAR2_402910 (Rbt51)	Rbt5/C4_00130W (h)	Rbt5 family	SP ^c^, GPI	+	+/+	+/+	+/+	+/+	+/+	+/+/+
CPAR2_300120 (Csa1)	Csa1/C7_00090C (o)	Rbt5 family	SP, GPI	−	−/−	−/−	−/+	−/+	−/−	+/+/+
CPAR2_108560 (Ecm33)	Ecm33/C1_03190C (o)	Ecm33 family	SP, GPI	+	+/+	+/+	+/+	+/+	+/+	+/+/+
Ecm331/CPAR2_100710	Ecm331/C5_02460C (o)	Ecm33 family	SP, GPI	+	+/+	+/+	+/+	+/+	+/+	+/+/+
CPAR2_603090 (Sun41)	Sun41/C6_00820W (o)	Sun family	SP, ASL	−	+/+	−/−	+/−	−/−	−/−	−/−/−
CPAR2_806490 (Pir1)	Pir1/C2_08870C (o)	Pir family	SP, 8 Pir repeats, ASL	+	+/+	+/+	+/+	+/+	+/+	+/+/+
Pga30/CPAR2_402000	Pga30/C4_04070C (o)	Pga30 family	SP, GPI	+	+/+	+/+	+/+	+/+	+/+	+/+/+
Rhd3/CPAR2_402010	Rhd3/C4_04050C (o)	Pga30 family	SP, GPI	−	+/+	+/+	−/−	−/−	−/−	−/−/−
CPAR2_603340 (Pga59)	Pga59/C4_02370C (o)	Pga59/Pga62 family	SP, GPI	+	−/−	−/−	−/−	+/+	+/+	−/−/−
CPAR2_503650 (Tos1)	Tos1/C3_01550C (o)	Unknown function	SP, ASL	+	+/−	+/−	+/−	+/+	+/+	+/−/+
CPAR2_301540 (Ssr1)	Ssr1/C7_00860W (o)	Unknown function	SP, GPI	+	+/+	+/+	+/+	+/+	+/+	+/+/+
CPAR2_200370 (Pga1)	Pga1/CR_10480W (o)	Unknown function	SP, GPI	−	−/−	−/−	+/−	+/−	+/−	−/−/−
CPAR2_400900 (Pga53)	Pga53/C4_01360W (o)	Unknown function	SP, GPI	+	−/−	−/−	−/+	−/−	+/+	−/−/−
CPAR2_701390	No hits	Unknown function	SP, GPI	−	+/+	+/+	−/+	−/−	−/−	−/−/+
CPAR2_805040	C1_10170W (o)	Unknown function	SP	−	−/−	+/+	−/−	−/−	−/−	−/+/+
CPAR2_403880	No hits	Unknown function	SP	−	+/+	−/−	−/−	−/−	−/−	−/−/−
CPAR2_405510 (Nce102)	NCE102/C3_04910C (o)	Unknown function	SP	−	−/−	−/−	−/−	−/−	−/−	+/+/+

^a^ St, Stationary phase; B_PS, polystyrol biofilm; B_S, silicone biofilm. ^b^ Total number of adhesin or non-adhesin peptides identified. ^c^ Protein identified (+) or not identified (−) in the sample. ^d^ Unclear GPI prediction for CPAR2_302140; CPAR2_402910 seemed to lack N-terminal ~23 aa.

## Data Availability

The data presented in this study are available in this paper and in the Supplementary Materials.

## Supplementary Materials

The following are available online at https://www.mdpi.com/article/10.3390/pathogens10040493/s1, Table S1: MS/MS identification of cell wall proteins in *C. parapsilosis*, Table S2: Semi-quantitative peptide counting of CW proteomic data from *C. parapsilosis* clinical isolates.
